# Coding-complete genome sequencing suggests that Newcastle disease virus challenge strain Herts’33 (IVMP) may represent a distinct genotype

**DOI:** 10.1007/s00705-019-04441-4

**Published:** 2019-11-08

**Authors:** Enikő Fehér, Ádám Bálint, Szilvia Marton, Krisztina Bali, Sándor Belák, Krisztián Bányai

**Affiliations:** 1 Institute for Veterinary Medical Research, Centre for Agricultural Research, Hungarian Academy of Sciences, Hungária krt. 21, Budapest, 1143 Hungary; 2 Veterinary Diagnostic Directorate, National Food Chain Safety Office, Budapest, Hungary; 3 Department of Biomedical Sciences and Veterinary Public Health (BVF), The OIE Collaborating Centre for the Biotechnology-Based Diagnosis of Infectious Diseases in Veterinary Medicine, Swedish University of Agricultural Sciences (SLU), Uppsala, Sweden

## Abstract

We determined the genomic sequence of a Newcastle disease virus (NDV) line obtained directly from the first NDV isolate, named Herts’33. This strain shared ≤ 90% nucleotide sequence identity with the NDV sequences available in the GenBank database, and formed a distinct branch in a phylogenetic tree. This branch may be considered to represent a separate NDV genotype. Our study indicates that investigation of the genomic sequences of old NDV strains that originated from the early outbreaks of Newcastle disease may alter the phylogenetic grouping of the NDV strains and provide data on the evolution of viral genomes over time.

Newcastle disease virus (NDV) (species *Avian orthoavulavirus 1*) belongs to the genus *Orthoavulavirus*, family *Paramyxoviridae*, order *Mononegavirales*. The enveloped virion has a single-stranded, negative-sense RNA genome with a length of ~ 15.2 kb [1, 2]. The viral genome is composed of six genes in the following order: 3’-NP-P-M-F-HN-L-5’ (letters designate the nucleoprotein (NP), the phosphoprotein (P), the matrix protein (M), the fusion protein (F), the hemagglutinin-neuraminidase (HN) and the RNA polymerase (L). Based on a number of criteria, the most recent genetic characterization classifies strains belonging to the species *Avian orthoavulavirus 1* into genotypes I to XXI and numerous subgenotypes within a subset of genotypes [1].

NDV infection may affect a wide range of wild and domestic avian species, causing serious losses during the epizootics [2]. The disease is controlled by vaccination of poultry, typically using live attenuated strains [2, 3]. For example, the viscerotropic Herts’33 strain was originally isolated from a field sample after an outbreak in 1933 in Hertfordshire (UK) and subsequently became an important strain for control and prevention of Newcastle disease (ND). The mesogenic H vaccine line was considered a derivate of the Herts’33 field strain and used as a potent live, attenuated vaccine on multiple continents, including Europe [4]. In addition, descendants of the original velogenic Herts’33 strain have been commonly used as challenge strains for testing ND vaccine efficacy.

With the introduction of molecular characterization methods, it has become possible to compare the genetic features of different descendants of Herts’33 and to analyze the homogeneities and differences in their genomes. In a 2003 paper, Czeglédi et al. [4] revealed that the descendant lines of Herts’33, including the vaccine strain H, differed in their F protein gene coding sequences, and three genetic groups were identified by phylogenetic analysis. It was hypothesized that the phylogenetic lineage that includes the Weybridge-origin Herts’33 strain might be a descendant of the isolate from the first ND case, while the Herts’33 lines that clustered phylogenetically with genotype IV sequences were of unknown origin. Interestingly, the vaccine strain H clustered with genotype III strains, including other vaccine strains (e.g., Mukteswar) [4].

In this study, we determined the complete coding sequence of a lineage Herts’33(W) [4] strain, which has been maintained in eggs and used as a challenge strain at the Institute for Veterinary Medicinal Products, Budapest, Hungary [4]. This strain, referred to here as Herts’33 (IVMP), was purchased in 1999 from the Veterinary Laboratories Agency, Weybridge, and then aliquoted in the Budapest institute. When starting the present study, an aliquot of the original seed was passaged once in eggs to obtain sufficient amount of material for genome sequencing. The viral RNA was extracted using a QIAamp Viral RNA Mini Kit (QIAGEN) and amplified by random RT-PCR as described elsewhere in detail [5]. The amplified cDNA was subjected to sequencing using the Ion Torrent PGM system (Life Technologies). Genome assembly was performed using CLC Genomic Workbench v7.0 (www.qiagenbioinformatics.com/products/clc-genomics-workbench/). Trimmed sequence reads were mapped onto reference NDV sequences to produce the consensus genome sequence of Herts’33 (IVMP).

The consensus sequence of Herts’33 (IVMP) consisted of 15,166 bases (accession no. MK674396) and lacked sequence information for a ~ 20 nt-long fragment in the 3’ end untranslated region. The genome organization was typical for NDV (Fig. 1), and the cleavage site in the F protein was typical for velogenic and mesogenic NDV strains (aa ^112^ RRQRR↓F^117^) [6, 7].

**Fig. 1:**
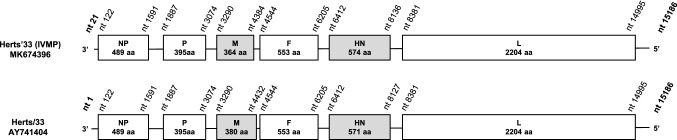
Schematic representation and comparison of the Herts’33 (IVMP) and Herts/33 genomes. NP, nucleocapsid protein; P, phosphoprotein; M, matrix protein; F, fusion glycoprotein; HN, hemagglutinin-neuraminidase protein; L, large or RNA-dependent RNA polymerase protein. The nt positions in Herts’33 (IVMP) were adjusted to those of the fully sequenced strain Herts/33

A BLAST search and phylogenetic analysis [8, 9] showed that the closest relative of Herts’33 (IVMP) was the genotype I (class II) strain Ulster/67 (accession no. AY562991), with 90.2% genome-wide identity. Regarding the complete F gene, we used the newly recommended method to calculate the average sequence identity among strains; as a result, the ≤ 88.3% nt sequence identity between the Herts’33 (IVMP) and the references fell below the cutoff value (i.e., 10% evolutionary distance) established for genotype discrimination for NDV strains [1, 10]. The topology of a phylogenetic tree based on the F gene also suggested that Herts’33 (IVMP) could be considered a separate genotype (Fig. 2; [1, 10]). Despite the molecular evidence that Herts’33 (IVMP) represents a unique genetic lineage, unfortunately, independent isolates related to this historic laboratory strain were not available for validation of our genetic classification [1, 10].

**Fig. 2:**
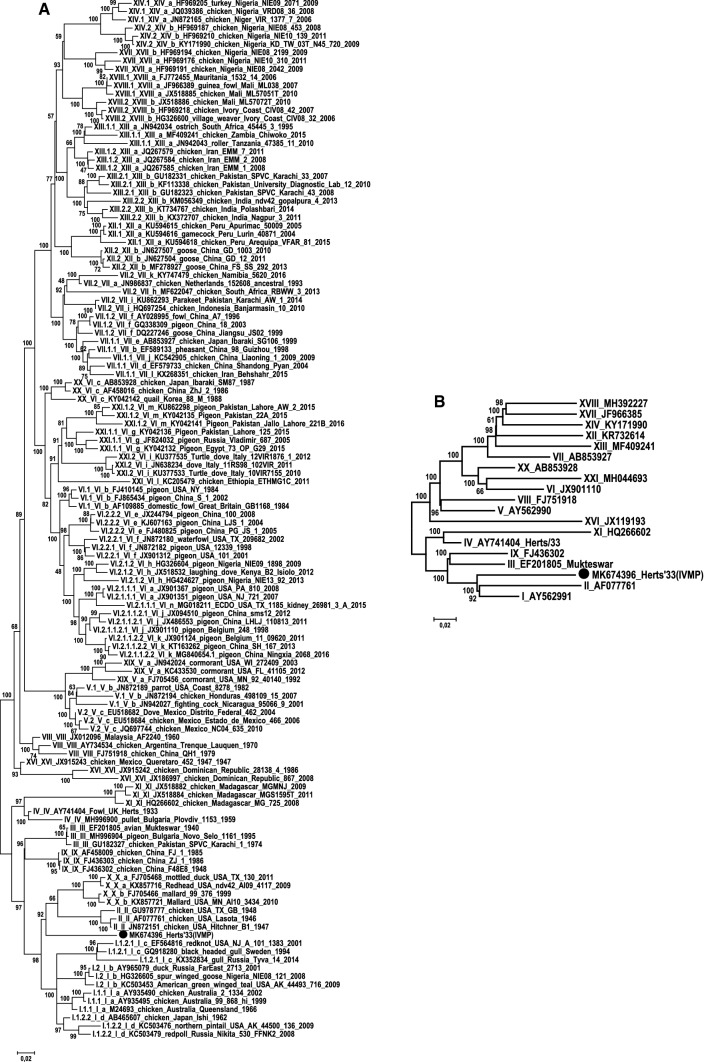
Phylogenetic analysis based on full-length fusion gene sequences (A) and representative complete genome sequences (B) using the maximum-likelihood method and the general time-reversible (G) model in MEGA6 software [9], with 100 bootstrap replicates. The Herts’33 (IVMP) sequence is indicated by a black dot

The strain Herts’33 (IVMP) and a genotype IV strain, Herts/33 (accession no. AY741404), shared 88.1% genome-wide and 85.7% F-gene-based nt sequence identity and clustered on different branches in the phylogenetic trees (Figs. 1, 2). Additional differences were seen when comparing the lengths of the deduced M and HN proteins (Fig. 1) [6]. The length of the M protein of Herts’33 (IVMP) was identical to that of most of the NDV isolates (364 aa) but shorter than the M protein of Herts/33 (380 aa). The HN protein of Herts’33 (IVMP) was 3 aa longer than the HN protein of other NDV strains (574 aa vs. 571 aa), including Herts/33 (Fig. 1), although additional size variation exists in other strains (e.g., Ulster/67, 616 aa). These data together imply a distinct evolutionary origin of the strains Herts’33 (IVMP) and Herts/33, a finding that corroborates earlier hypotheses [4].

Apparently, some reference NDV strains were maintained at various laboratories for decades without the precise knowledge of their genetic background. In this study, we provide genomic sequence data of an ‘old’ NDV challenge strain that likely represents an extinct genotype of NDV. This new genome sequence information will be useful to update schemes for classification of NDV isolates and to support more reliable evaluation of experimental data on the genetic diversity of various vaccine and challenge strains of NDV.
